# ChatGPT-polished scientific writing and AI detection: Cohorts, baselines, fairness

**DOI:** 10.1016/j.jdin.2025.10.017

**Published:** 2025-11-15

**Authors:** Wei Du, Shunsuke Koga

**Affiliations:** Department of Pathology and Laboratory Medicine, Hospital of the University of Pennsylvania, Philadelphia, Pennsylvania

**Keywords:** academic writing, artificial intelligence, ChatGPT, ethics, large language models, non-native English authors

*To the Editor:* We read the article by Wang et al, who report that ChatGPT-assisted polishing increases the chance that human-authored letters are flagged as artificial intelligence (AI)-generated. At baseline, 97% to 100% of originals were classified as human; however, after polishing, 75% to 85% were flagged as AI-generated, including 15% to 25% at high confidence.^1^ Prior correspondence has emphasized education on large language models strengths and limitations, and collaborative, transparent use to support non-native English authors.^2^^,^^3^ With this goal in mind, we outline 2 points to clarify interpretation and to suggest practical policy steps.

First, a transparent framework for group definition and sampling would help. Using U.S. institutional affiliation as a proxy for native English status risks misclassification. Since many U.S.-affiliated authors trained or grew up in non-English environments, affiliation alone may not capture language background. Additionally, the “non-English-speaking” cohort needs a clear rationale because only China, Korea, and Japan were included. A pre-specified sampling frame, such as based on submission volume, data availability, or a regional schema, would improve generalizability and mitigate concerns about selection bias.

Second, Figures 1 and 2 in their article suggest that detector outputs vary by language background and can place non-native authors at a disadvantage, even before the recent growth of generative AI, which raises fairness concerns for how these outputs are used.^1^ Figure 1 shows that the AI-generated probability for non-English-speaking authors in 2020 may exceed that for U.S. authors in 2024, a difference that predates the post-2022 rise in AI-assisted writing and points to a baseline pattern rather than a new effect. Figure 2 shows that at baseline, original letters from non-native authors receive fewer “human (high)” labels than those from U.S. authors, even though all are human-written, which is consistent with sensitivity to nativeness or fluency rather than AI-like features. This pattern aligns with prior work reporting that AI detectors can misclassify non-native English writing as AI-generated.^4^ Taken together, these patterns indicate that detector scores can conflate long-standing differences across language backgrounds with recent changes in tool use, and that using such scores as a proxy for authorship or quality can disadvantage non-native authors. The main question, therefore, is whether the content is correct and clear, not which tool was used to polish the language.

Efforts to detect AI-generated text evolve quickly and are easy to evade, and they do not solve the main problem. A better safeguard is rigorous peer review that checks claims, methods, and data. Because AI tools can produce inaccurate or fabricated citations, the process should also include strict verification that references exist and match the text. We support simple disclosure of AI assistance by authors. It is also reasonable that parts of peer review or editing may use AI for narrow tasks, such as reference checking and internal-consistency checks, while final judgments stay with human reviewers.

We agree with the authors’ fairness-focused message and, consistent with that goal, we emphasize transparency in AI use and rigorous content-based evaluation over attempts to label texts as AI- or human-written.^1^ We offer these observations to support equitable editorial practice.

## Declaration of generative AI and AI-assisted technologies in the writing process

During the preparation of this letter, Koga used ChatGPT (OpenAI, GPT-5 Pro) on August 17 and September 27, 2025, to improve readability and clarity. After using this tool, the authors reviewed and edited the content as needed and take full responsibility for the content of the publication.

## Conflicts of interest

None disclosed.
